# The experience of medical training and expectations regarding future medical practice of medical students in the Cuban-supported Medical School in Timor-Leste

**DOI:** 10.1186/s12960-015-0004-8

**Published:** 2015-03-28

**Authors:** Paulo Ferrinho, Ana C Valdes, Jorge Cabral

**Affiliations:** WHO Collaborating Centre on Health Workforce Policy and Planning, Centro de Malária e Outras Doenças Tropicais, Instituto de Higiene e Medicina Tropical, Universidade Nova de Lisboa, Lisbon, Portugal; School of General Medicine, Faculty of Medicine and Health Sciences, National University of Timor-Leste, Dili, Timor-Leste

**Keywords:** Timor-Leste, Medical training

## Abstract

**Background:**

The purpose of this paper is to describe and analyse the professional expectations and profile of medical students at the Cuban-supported School of General Medicine, in the Faculty of Medicine and Health Sciences of the National University of Timor-Leste.

**Methods:**

A piloted, standardized questionnaire, with closed- and open-ended questions, was distributed to registered medical students attending classes on the day of the survey. All data were analysed using SPSS. The statistical analysis is mostly descriptive.

**Results:**

Students decide to study medicine at an early age. Relatives and friends seem to have an especially important influence in encouraging, reinforcing and promoting the desire to be a doctor.

The degree of feminization of the student population is high.

Medical students are in general satisfied with the training received, though demanding improvements in terms of systems to support their studies and training (e.g. libraries, laboratories, access to computers and the Internet).

Medical students know that they will be needed in the public sector and that it would represent their opportunity to contribute to the public’s welfare. Nonetheless, they report that they expect to combine public sector practice with private work, probably, in order to improve their earnings. This may be explained by their expectations for salaries, which are much higher than the current level of public sector salaries.

A significant proportion of students are unsure about their future area of specialization. Of those that have determined their desired specialization, most intend to train as hospital specialists and to follow a hospital-based career. For many, specialization is equated with migration to study abroad. There are important differences between students at the start of their training compared with more advanced students.

**Contribution to the field:**

This paper gives an overview of student expectations for alignment with stated national human resources for health priorities for Timor-Leste.

## Background

Timor-Leste (T-L) is a small country which has embarked on a rapid process of developing its medical workforce in order to rebuild its health services weakened by a devastating civil war. This includes support from the Government of Cuba [1-6]. As part of its social, medical and scientific diplomacy [7,8], in the context of a broader cooperation strategy in the Pacific [2,3], Cuba is committed to training Timorese students in Cuba and, in addition, has sent a medical brigade to help train physicians at the School of General Medicine of the National University of Timor-Leste (SGM-UNTL), in Dili, the capital. This fast-track programme for the scaling-up of the medical workforce was analysed in a previous paper [9].

These doctors are expected to strengthen the network of health facilities of the national health system. This network suffers from a number of capacity shortcomings. Laboratory services in most community health centres are limited to non-instrumental techniques, and even the National Hospital in Dili struggles for basic imaging or oncology capacities. According to the Annual Report of the Ministry of Health of T-L for 2011, 46.7% of hospital admissions did not have a diagnosis, only 55% of notified malaria cases had a laboratory confirmation and approximately 37% of tuberculosis cases under treatment have dubious laboratory diagnosis. As these are the clinical units that support medical training, the situation described is likely to have impact on that training.

The syllabuses of the courses in T-L and Cuba are very similar. They share the same evolution from foundational medical sciences to clinical and community health practice, through 6 years, topped by a final (seventh) year of rotations (those students that started/completed their training in Cuba spent one additional—first—year for Spanish language training). These similarities have enabled the smooth transition from full courses in Cuba to combined programmes (the first 4 years in Cuba, followed by the last 3 in T-L) and finally the full course in T-L.

The T-L Government made huge efforts to increase the intake of students from rural areas into the SGM, at least for the initial batches. The students are regularly exposed to the limited technical capacity of the health system, during classes and rotations (accompanied by the stationed Cuban doctors). Physicians’ salaries were also improved from 2013 onwards. However, the limited technical capacities of the public health system combine with the harsh living conditions in this mostly rural (and rugged) country for a potentially rude blow at initial motivation, which may play a strong influence upon the intention to stay in these rural posts [10,11].

This paper provides a profile of medical students in the 2012–2013 academic year at the SGM of the Faculty of Medicine and Health Sciences (FMHS) of the UNTL. The purpose of the current investigation was the collection of demographic and social information, as well as data on their expectations and difficulties regarding their education and career plans. Our main hypothesis is that the students’ expectations may not become satisfied by the conditions of employment, work and life likely to be provided by the public health administration.

### The physician scaling-up strategy

Between 2003 and 2009, about 600 Timorese students were sent to Cuba for medical training, and the medical brigade initiated medical training in the SGM-UNTL, the National Hospital in Dili and also in district hospitals and community health centres across the country. The first group of eight medical graduates returned from Cuba in 2010. By 2012, students who started to study in 2007 in Cuba returned to T-L at the end of fourth year, to spend their last 2 years of training in the local Medical School. The local training in T-L has more modest targets, with yearly intakes of 45–50 students. The combined result of the training in Cuba and T-L will be the availability of approximately 800 new Timorese physicians between 2011 and 2015, while in 2010–2011, there were 233 physicians in T-L (of which only 64 were Timorese). This sudden massive influx of physicians will also change dramatically the composition of the health workforce overall: the 1300 nurses and midwives that existed in 2010 are growing at a pace of approximately 120 per year. The new physicians will be posted all over the country in health centres and hospitals of the public sector facilities of the national health system [9].

Graduate training for health professionals has been formally assigned exclusively to the UNTL, starting with the Faculty of Medicine and Health Sciences, which includes the School of General Medicine and the Nursing and Midwifery Schools. Though medical teaching is based on the presence of the Cuban medical brigade, teaching aids are scarce and basic medical equipment is also insufficient for practical training in most facilities [12].

## Methods

The methodology is similar to the one utilized in similar surveys in Angola, Guinea-Bissau and Mozambique [13-17]. A piloted, standardized questionnaire, with closed- and open-ended questions, was distributed to registered medical students attending classes on the day of the survey. There were no refusals to participate. No students on the third, fourth and fifth years were at the premises of the SGM on the days when the questionnaire was distributed (they were either preparing for exams or staying outside Dili). The questionnaires were distributed and collected by one of the authors during 2 days, in June, 2013. The students were not required to provide identifying information. The students had a previous explanation on the objectives of the survey, and the exact understanding of each and every question was also ensured by one of the researchers (the students have to struggle daily with various languages, and the questionnaire had been translated to Spanish, which is the language of their medical course training). All data were analysed using SPSS and frequencies (counts and percentages) are reported.

Permission to undertake the study was granted by the Dean of the Faculty of Medicine and Health Sciences of the UNTL, Dr João Martins. Ethical approval was given to the first application of this questionnaire in Mozambique. Since then, it has been applied in other countries [13,15-17]. The Ethics Committee of the Instituto de Higiene e Medicina Tropical (IHMT) does not require formal ethical approval for this type of research, though it has been notified according to internal regulations of the IHMT.

## Results

### Students’ demographics

In the 2 days of classes where the survey was distributed, a total of 150 students responded: 53 first year students, 24 second year students and 72 sixth year students. Programme year data was not available for one of the students.

Most of the students (57%) were born in Dili (52% of the first and second year students and 63% of the final year students), and about half received their primary (49%) and secondary (48%) school education in Dili where the medical school is located.

About half of the students were female (51%)—slightly more for the first and second year (53%) than for the final year (49%) students. Most (54%) had between 19 and 23 years of age, 34% were between 24 and 27 years old and 12% were aged 28 years or older. Of the 150 students, 9% were married and 9% studied and worked at the same time.

### The decision to study medicine

About half of the students (56%) took their decision to study medicine when they were aged 17 years or less and a quarter (27%) at the age of 12 or less years. All had decided to study medicine before age 25.

The reasons (more than one was possible) that students reported for choosing medicine as a profession were “to contribute to the welfare of the public” (73%), “self-realization and vocation” (15%), “because of the shortage of doctors in the country” (9%) and “social recognition/status” (3%). “Making money” was actually acknowledged as a reason only by one of the students. A larger percentage of senior students were more sensitive to reasons associated with shortages of medical staff in T-L (Table 1).

**Table 1:** **Reasons reported by students for choosing medicine as a profession (per year of training of the students)**

**Reasons**	**Year of study of medical students**		**Total**
	**1st and 2nd**	**6th**	
To contribute to the welfare of the public	59 (77%)	52 (69%)	111 (73%)
Self-realization and vocation	13 (17%)	10 (13%)	23 (15%)
Because of the shortage of doctors in the country	3 (4%)	11 (15%)	14 (9%)
Social recognition/status	2 (3%)	2 (3%)	4 (3%)

Only 31% of the students reported that they had relatives with some association with the health sector, and only 26% were related to doctors. These relatives were acknowledged as an important influence in their decision to choose medicine as their field of training.

### Academic performance

Academic performance seemed good in general with only two students acknowledging having failed one academic year and one student had one subject from a previous year that was still incomplete.

### Main difficulties reported

Although few students reported academic difficulties, only 41% of the students reported being satisfied with the academic support systems. The most frequent difficulties reported by the students were “lack of bibliography”, “incomplete facilities”, lack of transport, limited “access to computers, Internet … laboratories … and patients” and financial difficulties.

### Satisfaction with the academic education received

Most (93%) of the students were satisfied or very satisfied with the burden of lecturing and 99% with the course programme. Regarding the quality of the training received, 99% felt it was satisfying or very satisfying, while 64% felt it was too theoretical, although it prepared them to work as doctors anywhere in the world (78%) and as part of a team of health workers (93%).

### Expectations regarding their professional future and income

When asked about their intentions regarding the sectors where they would like to practise medicine after completing their medical education, the proportion of students interested in exclusive public sector work is high (77%) and, with one exception, private sector work is considered only in accumulation with the public sector appointment (23%). More senior students were more committed to exclusive public sector work than junior students, whereas combining public sector work with private practice was more appreciated among junior students (Table 2).

**Table 2:** **Intentions regarding the sectors of practice by seniority of the students**

**Sector of intended practice**	**Year of study of medical students**		**Total**
	**1st and 2nd**	**6th**	
Exclusively in the public sector	50 (68%)	61 (86%)	111 (77%)
Exclusively in the private sector	1 (1%)	0	1 (1%)
Combined public and private sectors	23 (31%)	10 (14%)	33 (23%)

Most (75%) would like to work in a hospital environment, 17% in a health centre and 7% at the governance of the health system, a distribution similar for junior and senior students.

As expected, 63% of the students born in Dili want to practise in Dili, versus 41% for those born outside Dili (52% of the total students surveyed want to practise medicine in Dili after graduation; this increases to 62% for junior students, as compared with 38% of more senior students).

Forty-one percent were not sure about specializing after graduation (61% of the junior students, compared with only 27% of the final year students). Of the 76 already considering specialization after graduation, 31% would opt for obstetrics and gynaecology (O&G) (24% and 39% for male and female students, respectively), 20% for several other surgical specialties, 12% for internal medicine, 12% for cardiology, 12% for paediatrics, 10% for other medical specialties and 3% for mental health. Though numbers are small, overall, O&G is the most attractive option for specialization among both junior and senior students (Table 3).

**Table 3:** **Preferred specialization per year of training**

**Preferred medical specialty**	**Year of study of medical students**		**Total**
	**1st and 2nd**	**6th**	
O&G	8 (29%)	16 (33%)	24 (31%)
Surgical	5 (18%)	10 (21%)	15 (20%)
Internal medicine	1 (4%)	8 (17%)	9 (12%)
Cardiology	4 (14%)	5 (10%)	9 (12%)
Paediatrics	6 (21%)	3 (6%)	9 (12%)
Other medical specialties	3 (11%)	5 (10%)	8 (10%)
Mental health	1 (4%)	1 (2%)	2 (3%)

The countries of choice for specialist training were Cuba (54%), Australia (14%), Indonesia (12%), Portugal (8%), T-L (6%) and Brazil (4%), with variations between senior and more junior students (Table 4).

**Table 4:** **Countries of choice for specialist training per year of study of medical students**

**Preferred country for specialization**	**Year of study of medical students**		**Total**
	**1st and 2nd**	**6th**	
Cuba	25 (34%)	53 (76%)	78 (54%)
Australia	16 (22%)	4 (6%)	20 (14%)
Indonesia	13 (18%)	3 (4%)	16 (11%)
Portugal	10 (13%)	2 (3%)	12 (8%)
T-L	6 (8%)	2 (3%)	8 (6%)
Other lusophone countries (Mozambique, Guinea-Bissau, Brazil)	3 (4%)	6 (8%)	9 (6%)

At a time when the starting salary of a doctor was about 600 US dollars a month, the students considered that a fair level of monthly income after graduation would be below 610 US dollars for 28%, 610–750 US dollars or less for 23%, 751–1000 US dollars for 33%, 1001–2000 US dollars for 13% and 2000 US dollars or over for 3%. Junior and female students have higher salary expectations than more senior and male students (Table 5).

**Table 5:** **Level of income considered fair for the first year of practice after graduation per sex and per year of study of medical students**

**Level of income considered fair for the first year of practice after graduation (US dollars)**	**Year of study of medical students**		**Sex of students**		**Total**
	**1st and 2nd**	**6th**	**Males**	**Females**	
<610	17 (22%)	25 (35%)	28 (38%)	14 (18%)	42 (28%)
610–749	20 (26%)	14 (19%)	14 (19%)	20 (26%)	34 (23%)
750–999	24 (31%)	24 (33%)	22 (30%)	27 (35%)	49 (33%)
	1 missing				
1000–1999	11 (14%)	9 (12%)	6 (8%)	14 (18%)	20 (13%)
≥2000	5 (6%)	0	3 (4%)	2 (2%)	5 (3%)

## Discussion

T-L is pursuing a bold strategy to recover from its acknowledged deficit in medical doctors. The decision to invest in the local training of more physicians is justified by the authorities of T-L by the need to be less dependent on foreigners and to attain a physician density comparable to regional averages [9].

About half the students in our sample were born in Dili and completed their secondary education in Dili, whereas the capital city comprises only 22% of the population of the country.

The objective of developing a “community-oriented” medical workforce, which draws on past experiences in Cuba, Israel, Australia, Ethiopia and South Africa [18,19], does not seem aligned with the expectation of the current students of, once graduated, working mostly in Dili and in hospitals. The preference for Dili seems accentuated in the younger generation of students, despite the fact that these younger students are more frequently born outside Dili than more senior students. This goes contrary to the expectation that selecting students from outside Dili would probably contribute to a higher percentage wanting to work outside the capital city [20,21].

Our results also match the findings from other surveys that most students would prefer to settle for hospital-based practice and work in the public sector [13-17,22].

Similar to the results in other Portuguese-speaking countries [13-17], motivations to choose the medical profession are linked to social commitment, “self-realization” and perceptions of opportunities related to the health labour market (shortage of doctors). More senior students seem more motivated by labour market issues than their junior colleagues.

Similar to findings from other surveys, i) there is a predominance of female students, a tendency accentuated among more junior students; ii) relatives in the health professions are an important influence in the choice of the medical profession; and iii) there is dissatisfaction with academic support systems [13-17,22].

Feminization of the student body is an important consideration with implications for career choices. Female doctors show a slight preference for future work in Dili rather than in rural and remote areas, as described in the literature [23-29].

Contrasting with data from other surveys [13-17,30-36] are the high levels of satisfaction with the programme and the workload and the high level of academic performance. The percentage with government bursaries is also much higher than in other medical training programmes [16]. It is however surprising that “financial difficulties” were frequently quoted among the Timorese students.

Similar to other studies [16,17,22], our data shows that at least 40% of the students were not sure of their future area of specialization, though the fact that half of our respondents were within the first 2 years of training may have influenced our results, as this subgroup had a much higher percentage of “undecided” (61%). This study in T-L confirms the little interest shown by medical students for post-graduate studies in basic sciences [35].

In many other developing countries, the critical step in the migration of medical graduates is the moment when they decide to obtain specialized training: a frequent individual decision is to look for it abroad [16,37,38], leading to a subsequent decision to stay in the receiving country [37]. A large majority of the students in T-L would like to obtain specialized training outside T-L, and the Ministry of Health of T-L estimates that 20% of the newly trained doctors will be lost to emigration [12]. Hence, T-L has already started a local medical post-graduation programme with support from Australian bilateral cooperation [12]. It is reassuring to note that the younger cohort of students in this survey show a greater tendency to select T-L as the venue for specialized training than sixth year students.

Salary expectations are highly inflated, a consideration that suggests that newly graduated doctors will look for other sources of income to complement their public sector salaries [16], a tendency most marked among junior and female students.

## Conclusions

Medical students are in general satisfied with the training received, though demanding improvements in terms of systems to support their studies and training—libraries, laboratories, access to computers and the Internet, among others.

Their future professional expectations are not aligned with the authorities’ intentions of developing a “community-oriented” medical workforce. Most students want to work in Dili and in hospitals. All of those with the intention of specializing want to further their careers in hospital-related specialties. Not a single student mentioned the intention of specializing either in public health or family medicine. Students’ satisfaction with the Cuban faculty is also reflected in the desire to further post-graduate studies in Cuba.

The concentration of Dili residents also counters the intention of achieving an equitable distribution of doctors in the country, as most students want to remain in Dili, the capital city, after graduation.

## Limitations

The major limitation in our data and its analysis is the absence of data from students in the mid-course years. This impacts particularly on the expectations for post-graduation. Another area where the absence of these students reduces the scope of the information gathered is on “Main difficulties reported” and “Satisfaction with the academic education received”, as students from fourth and fifth years would have been experienced with training conditions outside Dili.
